# A Decade of Non-Invasive Prenatal Testing (NIPT) for Chromosomal Abnormalities in Croatia: First National Monocentric Study to Inform Country’s Future Prenatal Care Strategy

**DOI:** 10.3390/genes15121590

**Published:** 2024-12-11

**Authors:** Petra Podobnik, Tomislav Meštrović, Aida Đorđević, Kristian Kurdija, Dženis Jelčić, Nina Ogrin, Ivan Bertović-Žunec, Beata Gebauer-Vuković, Grega Hočevar, Igor Lončar, Zlata Srebreniković, Petra Trobina, Marko Bitenc, Ivo Dumić-Čule

**Affiliations:** 1 Podobnik Special Hospital, Ul. Sveti Duh 112, 10000 Zagreb, Croatia; petra@podobnik.hr (P.P.);; 2 DA VINCI Polyclinic, Petrovaradinska ulica 110, 10000 Zagreb, Croatia; tmestrovic@davinci.hr; 3 University Centre Varaždin, University North, 42000 Varaždin, Croatia; 4 GenePlanet, Cesta na Poljane 3A, 1210 Ljubljana, Sloveniamarko.bitenc@geneplanet.com (M.B.)

**Keywords:** non-invasive prenatal testing, NIPT, cell-free foetal DNA, chromosomal abnormalities, aneuploidies, trisomy 13, trisomy 18, trisomy 21

## Abstract

Background: Chromosomal numerical and structural alterations are significant causes of various developmental disorders in foetuses. Non-invasive prenatal testing (NIPT) has emerged as an effective screening tool for detecting common aneuploidies, aiding in the identification of individuals who may require further diagnostic work-up. Methods: This retrospective, monocentric observational study evaluates the usage patterns, test choices, turnaround times (TAT), and outcomes of NIPT between 2013 and 2023 on a sample of 2431 pregnant women at a special hospital offering outpatient services and comprehensive gynaecological/obstetric inpatient care. We analysed the trends in NIPT usage, high-risk results, prior screening procedures, as well as factors such as age, gestational age and in vitro fertilisation (IVF) status. NIPT was performed using cell-free foetal DNA (cffDNA) extracted from maternal plasma, followed by library construction, sequencing and result analysis. The sequencing results were aligned with reference genomes, and z-scores were calculated to assess the likelihood of aneuploidy. Statistical significance was set at *p* < 0.05. Results: The average age of women undergoing NIPT decreased from 36.1 years in 2013 to 33.01 years in 2023 (*p* = 0.0287), and mean TAT dropped from 12.44 days in 2013 to 7.08 days in 2023 (*p* = 0.0121), with the most substantial reduction occurring between 2013 and 2019. The study identified a stable rate of women who underwent IVF seeking prenatal testing, with no statistically significant difference between the first half and the second half of the analysed period (*p* = 0.2659). Among high-risk results, there were 39 chromosomal abnormalities detected, most of them belonging to trisomy 21 (59%). Conclusions: Our findings demonstrate the increasing efficiency and accessibility of NIPT in prenatal care in Croatia, while the significant reduction in TAT and the decreasing age of women undergoing NIPT reflect enhanced operational practices and broader acceptance. Introducing NIPT into the public healthcare system in the Republic of Croatia could improve equitable access to advanced prenatal care and enhance pregnancy outcomes. Future advancements in technology and genetic counselling will further enhance its role, requiring careful attention to ethical and regulatory considerations.

## 1. Introduction

Chromosomal numerical and structural alterations are significant causes of various developmental disorders in the foetus, which are ideally detected early in pregnancy, during the first or second trimester, with the use of invasive and non-invasive procedures [1]. Aneuploidies are chromosomal abnormalities involving an atypical number of chromosomes, either extra or missing from the normal 23 pairs in humans, with incidence reaching up to one in every 160 live births [1,2]. While invasive methods to detect these conditions are indeed diagnostic, they carry a risk of abortion and thus are reserved for high-risk pregnancies with justified indications [3,4]. On the other hand, non-invasive methods or screening tests are designed to identify pregnant women at increased risk for the most common aneuploidies of chromosomes 21, 18, and 13, as well as the sex chromosomes X and Y [5,6]. When a high risk is detected, the pregnant woman is further referred for invasive prenatal diagnostics, such as chorionic villus sampling or amniocentesis, depending on the gestational age [6].

The advent of freely circulating foetal DNA (cell-free foetal DNA—cffDNA) in maternal blood in 1997 marked a significant milestone, enabling the development of new non-invasive prenatal screening tests [7]. By using polymerase chain reaction (PCR), Lo and co-authors initially determined the male sex of the foetus by amplifying markers on the Y chromosome [7]. This method was further enhanced through subsequent research, significantly increasing the sensitivity of detecting cffDNA for the most common aneuploidies [8,9,10]. Non-invasive prenatal testing (NIPT) analyses cffDNA released from the placenta, even though the term “foetal DNA” is commonly used in the literature [7]. It is important to state that this DNA is actually placental in origin, which is important for accurate result interpretation, and also that the methodology employed for cffDNA targeting, amplification, measurement and analysis can be substantially different between laboratories [4,5,11].

The effectiveness of NIPT, specifically the analysis of foetal cffDNA, is primarily due to its non-invasive nature, as it involves collecting peripheral venous blood from the mother, posing no risk to either the mother or the foetus—which makes it ideal for a screening procedure [12]. Additionally, it can be applied as early as the 9th week of pregnancy, much earlier than biochemical screening and invasive techniques [11]. The first commercial non-invasive prenatal test analysing cffDNA for screening the most common aneuploidies was introduced in 2011, while today, several methods can be used for analysing foetal cffDNA: targeted genome sequencing, massively parallel shotgun sequencing, targeted massively parallel sequencing, and single nucleotide polymorphism (SNP) sequencing [11,12,13].

In the Republic of Croatia, NIPT tests are not included in the national prenatal care strategy for chromosomal abnormalities but are available in a private healthcare setting with varying prices and screening panel options [14,15,16]. This situation contrasts with practices in countries like Norway, Sweden and Finland, where funding is provided for pregnant women at high risk for aneuploidies based on combined test results [15,17]. Denmark even went a step further by introducing NIPT for all pregnant women as the initial screening test [17]. In any case, the justification for incorporating NIPT in public funding is based on principles of autonomy and social justice, raising the question of whether the Republic of Croatia should adopt a similar approach in the near future.

To our knowledge, this study is the first attempt to analyse the age and pregnancy profile of the pregnant women population that undergoes NIPT testing in an 11-year period (2013–2023) upon its introduction in a single clinical centre in Croatia. It also aims to appraise the frequency of testing, test choices, any prior screening procedures, and a breakdown of high-risk results. We also evaluate parameters such as turnaround time (TAT), delays and other relevant factors linked to the testing procedure per se. By exploring these aspects, the study seeks to provide a comprehensive understanding of the current state of NIPT in Croatia and its impact on prenatal care, informing, in turn, potential future policies/practices to enhance prenatal screening and diagnostic services.

## 2. Materials and Methods

This retrospective, monocentric observational study encompassed 2431 pregnant women who visited Podobnik Special Hospital between 2013 and 2023. This institution stands out as the first and only private healthcare institution in Croatia offering a combination of outpatient services and comprehensive obstetric and gynaecological inpatient care, and it also introduced NIPT testing immediately after its introduction to the country. We aimed to assess the trends in the usage of NIPT in identifying trisomy 21 (Down syndrome), trisomy 18 (Edwards syndrome) and trisomy 13 (Patau syndrome), but also other chromosomal abnormalities and syndromes (including rarer conditions and microdeletions). NIPT was offered to the participants as either primary or secondary screening. To be eligible, participants had to be at least 18 years old and have either a singleton or twin pregnancy of ten weeks’ gestation or more. The study complied with the policies of the Helsinki Declaration. Every participant underwent detailed pre-test counselling to ensure they were fully informed about the procedure, while informed written consent was obtained from all participants prior to blood sampling.

Participants in the study were offered four types of NIPT options: Nifty Basic^®^, Nifty Standard^®^, Nifty Plus^®^, and Nifty Twins^®^ (Geneplanet LLC, 1000 Ljubljana, Slovenia). Nifty Basic^®^ provides a basic level of screening focused on the most common chromosomal abnormalities, such as trisomies 21, 18 and 13, can provide information on the foetal gender, and is suitable for singleton and twin pregnancies. Nifty Standard^®^ includes all the tests covered in Nifty Basic^®^ and extends to screen for sex chromosome aneuploidies (SCAs) and other specific chromosomal conditions for singleton pregnancies. Nifty Plus^®^ offers the most extensive screening, covering all conditions included in Nifty Basic^®^ and Nifty Standard^®^, as well as a broader range of chromosomal abnormalities and syndromes, including rarer conditions and microdeletions, and is suitable for singleton pregnancies. Lastly, Nifty Twins^®^ is specifically designed for twin pregnancies and is tailored to address the complexities of multiple gestations. These comprehensive options allowed participants to choose the most appropriate level of screening based on their specific needs and conditions of the pregnancy.

To perform these types of NIPT options, peripheral blood samples were collected from each pregnant woman in the collection tubes containing anticoagulants (Streck, La Vista, NE, USA). The tubes underwent centrifugation at 1600× *g* for 14 min within 96 h after collection to separate the plasma. The plasma was then transferred to microcentrifuge tubes and centrifuged again at 16,000× *g* for 10 min to ensure the removal of any remaining cellular debris. The cell-free plasma was subsequently stored at −80 °C until the DNA extraction process. Each plasma sample was frozen and thawed only a single time.

The cell-free foetal DNA (cffDNA) was then extracted from these samples and used for library construction, sequencing, and subsequent result analysis, following a previously described method with slight alterations [18]. Specifically, the process involved collecting 1.8 mL of plasma, which was then equally divided into three 2 mL nuclease-free centrifuge tubes for subsequent use. The plasma separation process included several quality control measures to ensure the integrity and accuracy of the samples. These measures included low-temperature treatment to preserve the plasma, determination of plasma haemolysis to assess the quality of the plasma, and verification of plasma volume to ensure sufficient quantity for accurate analysis.

The process of DNA library preparation involved end repair, adaptor ligation, and a series of PCR reactions. During end repair, the sample underwent a reaction that included extracted cffDNA, end repair buffer, and an end repair enzyme—performed at specific temperatures for designated durations. Following this, adaptor ligation was carried out, involving the DNA solution, ligation buffer, ligase and barcodes, also at controlled temperatures. After the ligation step, the DNA was purified, and PCR amplification was conducted to produce sufficient DNA for sequencing. The PCR involved a series of temperature cycles designed to amplify the adaptor-ligated DNA effectively.

To generate a library pool, the concentration of the library was measured using a Qubit fluorescence quantitative analyser (Life Technologies, Carlsbad, California, United States). The criteria for the measurements were blank control <0.6 ng/μL, negative and positive controls >2 ng/μL, and clinical samples >2 ng/μL. Quality control (QC) for sequencing required a DNA nanoball (DNB) concentration of ≥8 ng/μL and Lane’s QC (the latter referring to the quality control measures applied to each lane of a sequencing run), which included an average original data volume of ≥10 M, Q30 ≥ 85%, and a total quantity of ≥400 M. For information analysis QC, the criteria included an effective data volume of ≥3.5 M, GC mean (i.e., the average percentage of guanine and cytosine nucleotides in a DNA sequence) between 38% and 42%, Q30 ≥ 85%, original data volume ≥ 5.2 M, comparison rate between 70% and 100%, repetition rate between 0% and 5%, abnormal chromosome number between 0 and 3, and foetal concentration between 3.5% and 50%.

Throughout the experimental process, the temperature and humidity in the experimental areas were maintained at 19–25 °C and 20–80%, respectively, to preserve the integrity of biological samples and ensure consistent/reliable experimental results. Sequencing results were aligned with reference genomes, and z-scores were calculated for each chromosome using the Halos-NIFTY software, ver. 2.3.2.1011 (BGI-Shenzhen, Shenzhen, China). Of note, z-score is calculated for each chromosome to assess the likelihood of aneuploidy by comparing the observed quantity of DNA fragments from a specific chromosome in the test sample to the expected quantity based on a reference population. A z-score higher than 3 indicated a high risk of aneuploidy, 1.96 or lower indicated a low risk of aneuploidy, while a score higher than 1.96 but lower than 3 fell into the grey area, requiring the library to be reconstructed. All procedures were performed following the instructions provided by BGI. The findings were compiled into a comprehensive report that was provided to the healthcare provider. This report includes an assessment of the risk for the most common chromosomal abnormalities and any other significant genetic findings.

From these findings obtained through our analysis, we examined the types of high-risk results identified from 2013 to 2023. We have also analysed age and gestational age of pregnant women undergoing screening, their family history (with any previous chromosomal conditions), any prior screening procedures, and whether the pregnancy resulted from in vitro fertilisation (IVF). Additionally, we analysed the utilisation of different NIFTY tests, as well as metrics related to turnaround times (TAT) and the rates of delays, resamples, and no-calls over the analysis period. All data were presented as numbers or percentages. Student’s *t*-test was performed to evaluate statistical significance where applicable and a *p*-value of <0.05 (two-tailed) was considered statistically significant. R software version 4.3.0 was used for statistical analyses [19].

## 3. Results

A total of 2431 pregnant women who visited the clinic between 2013 and 2023 underwent NIPT. The average age of all study participants was 35.4, with a minimum age of 18 and a maximum age of 49 (Table 1). The average gestational age was 12 + 4 weeks, while the median was 12 + 2 weeks (with minimum gestational age of 10 + 1 weeks and maximum of 19 + 3 weeks). Through the analysis period (i.e., from 2013 to 2023), minimum and maximum ages varied slightly but did not show a consistent trend, while the average age of patients showed a decreasing trend from 36.1 years in 2013 to 33.01 years in 2023. Among the total number of study participants, 305 women, accounting for 12.5%, achieved pregnancy through in vitro fertilisation (IVF)—with observable changes in this trend over the period from 2013 to 2023 (Table 2). More specifically, there was a generally upward trend from 2013 to 2021, with some stabilisation in the middle years and then a decline in 2022 and 2023 (suggesting possible external factors or changes in participant demographics and/or IVF protocols).

To assess whether there has been a significant change over the years in the average age and the number of women who became pregnant through IVF, a *t*-test was performed comparing the average age in the first half of the period (2013–2017) vs. the second half (2018–2023). The change in the average age from the first half of the period (2013–2017) to the second half (2018–2023) was statistically significant (t = 2.6015, *p* = 0.0287). There was no statistically significant difference in the number of IVF-pregnant women seeking prenatal testing between the first half (2013–2017) and the second half (2018–2023) of the analysed period (t = −1.1863, *p* = 0.2659).

The data on prior screening indicates that 221 (or 9.09%) had undergone prior screening, while the remainder of the women had not. Among those who had prior screening, the majority underwent combined ultrasound (nuchal translucency measurement, crown-rump length) and biochemistry (β-HCG, pregnancy-associated plasma protein-A) screening (combined with mother’s age) in the first trimester, accounting for 144 cases (or 65.16%) This was followed by 47 cases (21.27%) classified under other, unspecified types of screenings. The first and second trimester integrated test—NT measurement and blood tests for the first trimester and quad screen that looks at α-fetoprotein, β-hCG, unconjugated estriol and inhibin A—was performed in 13 cases (5.88%). Finally, only second trimester screening was performed in 11 cases (4.98%), whereas only biochemistry screening in the first trimester was the least common, with only 6 cases (2.71%). In our sample, 39 women reported previous chromosomal conditions, 19 women had a family history of genetic diseases, while 8 of them were carriers of a genetic condition.

Table 3 summarises the distribution of various Nifty test types conducted from 2013 to 2023. Over this period, Nifty Plus^®^ was the most commonly performed test, accounting for 72.07% of all tests (1752 out of 2431), followed by Nifty Standard^®^ at 24.52% (596 tests), with Nifty Basic^®^ and Nifty Twins^®^ being much less frequently used, representing 2.63% (64 tests) and 0.78% (19 tests) of the total, respectively. In the period from 2017 to 2019, the introduction of new partners in the Zagreb area offering NIPT tests likely contributed to a smaller increase in the number of tests performed at the analysed institution. Additionally, in 2020, there was a noticeable shift with the proportion of Nifty Plus^®^ tests decreasing and the number of Nifty Standard^®^ tests increasing, which is attributed to greater awareness and accessibility of prenatal testing among a broader socioeconomic population. Table 4 provides a comprehensive overview of the outcomes for high-risk prenatal test results obtained using Nifty^®^ tests, spanning various chromosomal abnormalities.

Table 5 summarises the occurrences of delays, resamples, and no calls (or test failures) for Nifty^®^ tests from 2013 to 2023. Overall, there were 87 instances of delays, accounting for 3.58% of the total tests. The highest delay rates occurred in 2020 and 2023, with 17 delays each year, representing 5.5% and 6.4% of the tests, respectively. Repeat blood sampling accounted for 25 in total, making up 1.03% of all tests, with the highest rate in 2023 at 2.2%. The number of test failures was minimal, with only four instances accounting for 0.16% of the total tests. This was scattered sporadically across the years with no occurrences in many years.

The mean TAT was 8.14 days, with a median of 8.00 days (Table 6). The mean TAT decreased significantly from 12.44 days in 2013 to 7.08 days in 2023, with the most substantial reduction occurring between 2013 and 2019 (Table 5). To assess the significance of this reduction, we compared the TAT for the first half of the period (2013–2017) with the second half (2018–2023) using a *t*-test. The analysis revealed a statistically significant difference between these two periods (t = 3.1311, *p* = 0.0121), indicating improved efficiency in the testing processes over time.

## 4. Discussion

In this study, we aimed to evaluate NIPT usage patterns, test choices, turnaround times and outcomes of prenatal screening for detecting chromosomal abnormalities over the last decade at a single institution in Croatia, which gave us valuable longitudinal data. We see it as a first step before pursuing more detailed clinical laboratory correlation studies in the future. However, these insights into the changing landscape of prenatal screening, as the demonstration of popularity, usability and need, can already be used to inform future prenatal care strategies in the country. The study’s large sample size of 2431 pregnant women who visited the clinic between 2013 and 2023 shed light on many different trends.

Considering the decreasing cost of high-throughput sequencing in recent years, the practicality and growth in usage in clinical settings NIPT is not surprising. Throughout our study time frame, we observed significant changes in the average age of women undergoing NIPT, decreasing from 36.1 years in 2013 to 33.01 years in 2023. This reduction may be attributed to various factors, but primarily to increased awareness and accessibility of NIPT [12,16], as we would not expect significant changes in the demographic profile of pregnant women. However, we have to emphasise notable changes occurring in the services offered by the clinic during this period, which entails new or enhanced services designed to attract a younger demographic and more comprehensive prenatal care options.

On the other hand, despite advancements in reproductive technology, the number of women who became pregnant through IVF and sought prenatal testing remained stable over the study period. One notable reason is that medical protocols and guidelines for prenatal testing in IVF pregnancies have been well-established and standardised [20,21], while the awareness and acceptance of prenatal testing among these women have been traditionally high [22]. In addition, the demographics of women seeking IVF treatments, such as age and health conditions, have not experienced significant shifts during the observed time span.

Prior to deciding on NIPT, 9.09% of women in our study sample have also undergone other screening procedures. The most common prior screening method was the combined first trimester ultrasound and biochemistry screening, followed by various other tests. The data also revealed that a subset of women reported a family history of genetic diseases or were carriers of genetic conditions, highlighting the importance of comprehensive pre-test counselling and genetic counselling services in prenatal care [23]. This is significant because one study revealed that while the majority of women (76%) believed that screening for a wide range of conditions would reduce suffering, 39% were concerned that it would present couples with complex choices that are hard to understand [24]. Likewise, “additional findings” beyond trisomy 21, 18, or 13 can lead to high anxiety and severe distress in 15.5 and 7.5% of women, respectively [25].

A statistically significant decrease in TAT in our study suggests that operational efficiencies, improved workflow processes and better management practices have been successfully implemented over the years, leading to faster turnaround times for prenatal testing. A similar trend has been observed in Denmark and other countries [26]. This improvement is needed in a modern healthcare system for the timely delivery of critical information to expectant parents and healthcare providers if our end goal is better prenatal care and decision-making. Of course, operational efficiency can be maintained and further enhanced by ensuring staff training and maintaining equipment performance [11].

A recent correlation analysis from China revealed that the introduction of NIPT as a publicly funded project by the Changsha government in Hunan Province resulted in increased uptake of the test and a significant reduction in aneuploid live births [5]. The data demonstrated that NIPT had high positive predictive value, sensitivity, and specificity for detecting T21, T18, and T13, thus playing a crucial role in the prevention and control of birth defects [5]. Notably, the study achieved positive screening rates of approximately two per thousand for T21, one per thousand for T18, and one per thousand for T13. As a result, NIPT led to 8.98 fewer live-born cases of T21 per 10,000 births, 1.64 fewer cases of T18 per 10,000 births, and 0.05 fewer cases of T13 per 10,000 births [5]. Such detection rates are comparable to our findings (Table 4).

For NIPT, the occurrence of false positive and false negative cases is inevitable [6,27,28], and our study also shows a low number of three false positives and one false negative case. Unlike conventional screening, false positives in NIPT are due to biological factors rather than technical errors [11]. The primary cause of discrepancies in karyotyping results is that circulating cell-free foetal DNA (cffDNA) originates from placental trophoblast cells and a small number of foetal cells, which may not accurately represent the foetus [11,27]. Other possible reasons for false positivity include the use of assisted reproductive technology, as well as issues in data analysis and annotation [27]. There are also factors such as foetal or placental mosaicism, which can occur due to meiotic errors and may be influenced by maternal age, as well as conditions like a vanished twin or maternal cancer [27,28,29,30], potentially leading to the occurrence of false positives. Additionally, the positive predictive value of NIPT is lower for certain conditions, such as monosomy X, and while placental mosaicism can be detected by NIPT, further research is needed to confirm its sensitivity [29,30].

There is also a Cochrane review that evaluated the accuracy of genomics-based non-invasive prenatal testing (gNIPT) for detecting common foetal aneuploidies in pregnant women based on their prior risk levels [6]. Specifically, the authors compared the diagnostic performance of two next-generation sequencing methods: massively parallel shotgun sequencing and targeted massively parallel sequencing, both of which analyse cffDNA in maternal plasma. The results indicate that these two techniques have similar clinical sensitivity and specificity for detecting foetal T21, T18, T13, and sex chromosome aneuploidy [6]. Furthermore, in high-risk pregnancies, these gNIPT methods demonstrated high accuracy for detecting the three major trisomies (i.e., T21, T18, and T13), with sensitivities ranging from 95.8% to 99.7% (depending on the specific trisomy), and specificities exceeding 99% [6].

The primary causes of assay failure in NIPT can include the failure of samples to pass quality control checks, insufficient amounts of ccfDNA, as well as low foetal fraction DNA [31]. Maternal factors such as higher maternal weight or obesity, increased blood pressure, as well as conditions like preeclampsia or autoimmune diseases can also contribute to NIPT failure [31]. In our study, the failure rate that necessitated resampling was 1.03%, which is lower than the 1.46% reported in a similar study conducted in Luxembourg [32]. This suggests a relatively robust performance of our assay protocol. Additionally, a Cochrane review has shown that failure rates can vary significantly depending on the method used. For massively parallel shotgun sequencing, failure rates ranged from 0% to 25%, while for targeted massively parallel sequencing, the rates ranged from 0.8% to 7.5% [6]. These variations stress the importance of methodological differences and the need for stringent quality control measures to minimise potential assay failures.

Taking our findings into account, we believe the field of healthcare is ready for the widespread introduction of this type of screening. Incorporating NIPT into public healthcare could enhance equitable access to advanced prenatal care, potentially reducing the prevalence of undetected chromosomal abnormalities and improving overall pregnancy outcomes [15]. We anticipate that technological advancements will lead to a reduction in the costs associated with conducting NIPT, thereby increasing its accessibility and utilisation. This cost reduction could also potentially pave the way for partial or full funding by governmental health agencies.

Alongside technological improvements, there is a critical need to focus on the education of both NIPT service providers and patients [33,34]. Healthcare personnel delivering NIPT services must be well-trained to clearly and effectively communicate the benefits and limitations of the test, as well as the implications of the results [34]. On the patient side, it is essential that they understand that NIPT is a screening tool and not a definitive diagnostic method, so any risks identified through NIPT should be confirmed via invasive prenatal diagnostic procedures [35]. Additionally, due to the nature of screening tests, there is a possibility of false positive and false negative results, which can arise from factors such as placental mosaicism, the demise of one twin, maternal sex chromosome aneuploidy, among other conditions (as already discussed) [27,28,29,30].

Therefore, before widespread introduction within the national strategy, the aforementioned key limitations should be considered, but also some other concerns. The increased use of commercial NIPT tests in low-risk populations inevitably results in potential false positive results due to low positive predictive value in these groups [14,36], but also due to other causes that were previously mentioned. Additionally, cffDNA derived from the placenta can exhibit mosaicism in 1–2% of cases between the 10th and 12th gestational weeks, leading to false positive and false negative results [37,38].

Importantly, during embryonic development, trisomy in trophoblast tissue may spontaneously regress in embryonic tissue, resulting in a euploid foetus [39,40]. In multiple pregnancies, NIPT is less ideal due to reduced isolated foetal DNA per foetus, increasing the risk of inaccurate results [41,42]. NIPT also cannot detect anatomical malformations, neural tube defects, or ventral wall defects [43,44]. Clearly, genetic counselling is essential before testing to explain genetic risks NIPT cannot detect and to clarify the limitations of the method, including the possibility of either false positive or false negative results [45]. These may be some of the reasons why no country or region has fully replaced traditional prenatal screening with NIPT, as highlighted in a recent scoping review [46].

Future advancements in NIPT technology are expected to enable whole-genome sequencing of the foetus, which will provide comprehensive information about the unborn child [13,47]. This could include carrier status for genetic conditions and predisposition to diseases that manifest later in life. Such advancements would significantly expand the scope of prenatal screening, offering a deeper understanding of the foetal genetic profile—however, it raises ethical and social considerations such as privacy and potential discrimination [48]. The expanded scope of prenatal care could facilitate early interventions for genetic conditions but also introduces the controversial ability to detect non-clinical traits (such as athletic abilities and hair/eye colour) [15,48]. Public health policy will need to adapt to manage these implications, ensuring equitable access and developing guidelines. Likewise, healthcare infrastructure and provider training require significant upgrades to support advanced technology and precise result interpretation.

One of the prerequisites is also improved regulation of NIPT, which makes establishing national guidelines and standards that ensure uniform protocols and quality assurance measures essential [49]. Government oversight through a dedicated regulatory body and accreditation programs for providers and laboratories can enforce these standards. Comprehensive education and training for healthcare providers, along with robust patient education and counselling, are crucial for informed decision-making [13]. Ethical and privacy protections, including strict informed consent guidelines and data privacy regulations, must be implemented. Additionally, subsidising NIPT for high-risk pregnancies and integrating it into publicly funded prenatal care can enhance equitable access [50]. Continuous research and feedback mechanisms should be promoted to monitor and improve NIPT services, while ethical review boards and public engagement can address broader ethical and social implications.

Our study has several limitations that should be acknowledged. We did not include a cost–benefit analysis or detailed statistical metrics such as positivity rate, positive predictive value, sensitivity or specificity, as it was primarily a retrospective, observational study focused on tracking trends in NIPT utilisation within a single private healthcare setting in Croatia. Conducting such analyses would require more detailed datasets and a broader study design, including confirmatory tests and cost data. Furthermore, due to the monocentric nature of the study, the findings may not be generalisable to other healthcare settings or populations. Moreover, using the data from a private healthcare facility may introduce selection bias, as the sample may not represent the broader population, particularly those who cannot afford this type of screening. The study also did not analyse the distribution of high-risk results over time to identify any trends or changes in frequency. In addition to monitoring TAT, other parameters (such as deeper insights into false positive and false negative rates) would be much more informative. We also did not investigate the actual underlying causes of the false positive and false negative NIPT results or determine their correlation with clinical outcomes. Future studies will need to incorporate more clinical laboratory correlations and directly compare different methodologies within the same patient cohort, but also compare high-risk and low-risk pregnancies to ascertain real clinical value.

## 5. Conclusions

This study highlights the significant trends and outcomes of NIPT over the past decade in a single centre in Croatia, underscoring its growing importance in prenatal care. Although we need to be cautious when interpreting the results in a broader context, our findings indicate a notable decrease in the average age of women undergoing NIPT, improvements in turnaround times, and a stable usage pattern among IVF pregnancies. Despite inevitable false positives and negatives, the high sensitivity and specificity of NIPT for detecting trisomies and other chromosomal abnormalities affirm its clinical utility. The integration of NIPT into public healthcare systems appears both feasible and beneficial; for the Republic of Croatia, that step could enhance equitable access to advanced prenatal care, reduce the prevalence of undetected chromosomal abnormalities, and improve overall pregnancy outcomes. Future advancements in NIPT technology and comprehensive genetic counselling will further enhance its role in prenatal screening, promoting, in turn, informed decision-making. However, continuous evaluation, ethical considerations and regulatory oversight are essential considerations to maximise the benefits and address potential challenges associated with widespread NIPT implementation.

## Figures and Tables

**Table 1:** A yearly age breakdown of study participants, with average, minimum and maximum age.

Year	Average Age	Minimum	Maximum
2013	36.1	20	43
2014	35.5	19	48
2015	35.3	22	44
2016	34.7	20	45
2017	34.1	20	46
2018	33.8	22	49
2019	34.2	19	43
2020	33.4	22	47
2021	32.98	20	43
2022	33.01	18	46
2023	33.01	21	45
Overall	35.4	18	49

**Table 2:** A yearly early breakdown of study participants who achieved pregnancy through in vitro fertilisation (IVF), shown as absolute numbers and percentages.

Year	Yes	No	Total
2013	1 (1.2%)	85 (98.8%)	86
2014	8 (6.5%)	116 (93.5%)	124
2015	19 (13.5%)	122 (86.5%)	141
2016	30 (13.9%)	186 (86.1%)	216
2017	32 (12.1%)	232 (87.9%)	264
2018	30 (11.3%)	236 (88.7%)	266
2019	41 (16.0%)	216 (84.0%)	257
2020	37 (12.1%)	270 (87.9%)	307
2021	58 (21.2%)	215 (78.8%)	273
2022	34 (12.4%)	240 (87.6%)	274
2023	15 (6.7%)	208 (93.3%)	223
Overall	305 (12.5%)	2126 (87.5%)	2431

**Table 3:** A yearly breakdown of Nifty^®^ NIPT test types conducted from 2013 to 2023 in the study sample, shown as absolute numbers and percentages.

Year	Nifty Basic^®^	Nifty Standard^®^	Nifty Plus^®^	Nifty Twins^®^	Total
2013	0 (0.0%)	86 (100.0%)	0 (0.0%)	0 (0.0%)	86
2014	0 (0.0%)	122 (98.4%)	0 (0.0%)	2 (1.6%)	124
2015	0 (0.0%)	7 (5.0%)	133 (94.3%)	1 (0.7%)	141
2016	0 (0.0%)	5 (2.3%)	207 (95.8%)	4 (1.9%)	216
2017	10 (3.8%)	35 (13.3%)	212 (80.3%)	7 (2.7%)	264
2018	7 (2.6%)	46 (17.3%)	210 (78.9%)	3 (1.1%)	266
2019	9 (3.5%)	46 (17.9%)	201 (78.2%)	1 (0.4%)	257
2020	13 (4.2%)	75 (24.4%)	219 (71.3%)	0 (0.0%)	307
2021	11 (4.0%)	47 (17.2%)	214 (78.4%)	1 (0.4%)	273
2022	10 (3.6%)	60 (21.9%)	204 (74.5%)	0 (0.0%)	274
2023	4 (1.8%)	67 (30.0%)	152 (68.2%)	0 (0.0%)	223
Overall	64 (2.63%)	596 (24.52%)	1752 (72.07%)	19 (0.78%)	2431

**Table 4:** A breakdown of outcomes for high-risk prenatal test results obtained with NIPT.

Chromosomal Abnormality	Total	Confirmed	No Info	False Positive	False Negative
High Risk T21	23	22	1	0	1
High Risk T18	2	1	1	0	0
High Risk T16	1	0	1	0	0
High Risk T13	1	1	0	0	0
High risk 45,X	3	1	2	0	0
High Risk XXX	2	2	0	0	0
High Risk XXY	2	1	1	0	0
High Risk T5 (mosaic T5)	1	1	0	0	0
High Risk T2 (incidental finding)	1	0	0	1	0
High Risk Cri Du Chat	1	0	0	1	0
High Risk, incidental finding dup (9p24.3–p13.1, 38.68 M)	1	1	0	0	0
High Risk Other Complex Abnormality	1	0	0	1	0

**Table 5:** Delays, resamples and no calls for NIPT over the study period (2013–2023).

Year	Delay	Resample	No Call
2013	0 (0.0%)	1 (1.2%)	0 (0.0%)
2014	0 (0.0%)	1 (0.8%)	0 (0.0%)
2015	2 (1.4%)	2 (1.4%)	0 (0.0%)
2016	6 (2.8%)	2 (0.9%)	1 (0.5%)
2017	8 (3.0%)	3 (1.1%)	1 (0.4%)
2018	7 (2.6%)	2 (0.8%)	0 (0.0%)
2019	8 (3.1%)	4 (1.6%)	1 (0.4%)
2020	17 (5.5%)	1 (0.3%)	0 (0.0%)
2021	8 (2.9%)	4 (1.5%)	1 (0.4%)
2022	14 (5.1%)	0 (0.0%)	0 (0.0%)
2023	17 (6.4%)	5 (2.2%)	0 (0.0%)
Total	87 (3.58%)	25 (1.03%)	4 (0.16%)

**Table 6:** The change in turnaround time (TAT) over the study period (2013–2023).

Year	TAT	TAT (Days, Rounded)
2013	12.44	12
2014	10.47	10
2015	8.44	8
2016	8.10	8
2017	8.00	8
2018	7.55	8
2019	6.58	7
2020	6.87	7
2021	7.08	7
2022	6.88	7
2023	7.08	7

## Data Availability

The original dataset is available upon reasonable request from the authors. The data are not publicly available due to the privacy of the participants.
